# *Brucella abortus* in Kazakhstan, population structure and comparison with worldwide genetic diversity

**DOI:** 10.3389/fmicb.2023.1106994

**Published:** 2023-03-22

**Authors:** Alexandr Shevtsov, Axel Cloeckaert, Kalysh Berdimuratova, Elena Shevtsova, Alexandr V. Shustov, Asylulan Amirgazin, Talgat Karibayev, Dinara Kamalova, Michel S. Zygmunt, Yerlan Ramanculov, Gilles Vergnaud

**Affiliations:** ^1^National Center for Biotechnology, Astana, Kazakhstan; ^2^INRAE, UMR ISP, Université de Tours, Nouzilly, France; ^3^National Reference Center for Veterinary, Astana, Kazakhstan; ^4^School of Sciences and Humanities, Nazarbayev University, Astana, Kazakhstan; ^5^Université Paris-Saclay, CEA, CNRS, Institute for Integrative Biology of the Cell (I2BC), Gif-sur-Yvette, France

**Keywords:** *Brucella abortus*, genetic diversity, WGS, SNP, genotyping, epidemiology

## Abstract

*Brucella abortus* is the main causative agent of brucellosis in cattle, leading to severe economic consequences in agriculture and affecting public health. The zoonotic nature of the infection increases the need to control the spread and dynamics of outbreaks in animals with the incorporation of high resolution genotyping techniques. Based on such methods, *B. abortus* is currently divided into three clades, A, B, and C. The latter includes subclades C1 and C2. This study presents the results of whole-genome sequencing of 49 *B. abortus* strains isolated in Kazakhstan between 1947 and 2015 and of 36 *B. abortus* strains of various geographic origins isolated from 1940 to 2004. *In silico* Multiple Locus Sequence Typing (MLST) allowed to assign strains from Kazakhstan to subclades C1 and to a much lower extend C2. Whole-genome Single-Nucleotide Polymorphism (wgSNP) analysis of the 46 strains of subclade C1 with strains of worldwide origins showed clustering with strains from neighboring countries, mostly North Caucasia, Western Russia, but also Siberia, China, and Mongolia. One of the three Kazakhstan strains assigned to subclade C2 matched the *B. abortus* S19 vaccine strain used in cattle, the other two were genetically close to the 104 M vaccine strain. Bayesian phylodynamic analysis dated the introduction of *B. abortus* subclade C1 into Kazakhstan to the 19th and early 20th centuries. We discuss this observation in view of the history of population migrations from Russia to the Kazakhstan steppes.

## 1. Introduction

Brucellosis is a bacterial zoonotic disease caused by species belonging to the genus *Brucella* and results in high economic impact (McDermott et al., 2013). *Brucella* spp. may be transmitted to humans resulting in a severe disease requiring a specific and long-term antibiotic treatment with significant burden to public health systems (Franc et al., 2018). The genus *Brucella* currently contains 12 validly published species (Olsen and Palmer, 2014; Whatmore and Foster, 2021; Occhialini et al., 2022). *Brucella melitensis*, *Brucella abortus*, *B. suis*, *B. ovis*, *B. neotomae,* and *B. canis* are often referred to as “classical” *Brucella* species in the literature. These have been identified between 1887 and 1968 and were differentiated on the basis of phenotypic traits and host preference (Buddle, 1956; Stoenner and Lackman, 1957; Carmichael and Bruner, 1968; Moreno et al., 2002). Since 2007, the wider use of genetic methods of identification and differentiation has led to the identification of *B. ceti*, *B. pinnipedialis*, *B. microti*, *B. inopinata*, *B. papionis,* and *B. vulpis*, which also preserve the tradition of naming the species in accordance with their original host (with the exception of *B. inopinata* isolated from a breast implant; Foster et al., 2007; Scholz et al., 2008, 2010; Whatmore et al., 2014; Scholz et al., 2016). The use of molecular methods made it possible to identify other potential new species recovered not only from mammals but also from amphibians, reptiles, and fish (Soler-Llorens et al., 2016; Al Dahouk et al., 2017; Eisenberg et al., 2017; Mühldorfer et al., 2017; Eisenberg et al., 2020), leading to further expansion of our knowledge of the genus *Brucella*. Based on whole-genome comparisons, a merge of the *Ochrobactrum* and *Brucella* genus was recently proposed (Hordt et al., 2020; Leclercq et al., 2020). Nonetheless, the greatest impact on public health and livestock infections around the globe have to date only been caused by *B. melitensis, B. abortus* and *B. suis* (Godfroid et al., 2011; Franc et al., 2018). An increase of human cases due to *B. canis* is currently suspected (Hensel et al., 2018; Zhou et al., 2020).

The majority of brucellosis cases are reported in the Mediterranean countries, South and Central America, Africa, Asia, Arabian Peninsula, Indian subcontinent, Eastern Europe, and the Middle East (Pappas et al., 2006; Nicoletti, 2010). Over the past decade, there has been a decline in incidence in many regions previously considered to be highly endemic, but also new reservoirs have been identified, such as in Africa and the Middle East, possibly resulting from a better implementation of diagnostic methods (Wang and Jiang, 2020). The real incidence of the disease in the mentioned regions is most probably largely underestimated because registration is based on passively collected data (Dean et al., 2012).

In Kazakhstan, brucellosis remains a major livestock and public health problem. More than 1,300 cases of human brucellosis are registered annually corresponding to an incidence of 7.6 per 100,000 inhabitants. Seropositivity to *Brucella* antigens in cattle and small cattle is 0.6% and 0.4%, respectively (Charypkhan and Ruegg, 2022).

The high zoonotic potential, re-emergence of the infection in previously disease-free regions, and identification of new reservoirs underscore the need for modern molecular epidemiology approaches such as genotyping to trace source reservoirs and paths of introduction. For *Brucella* genotyping at subspecies level, two methods are most widely used, Multilocus Sequence Typing (MLST) and Multiple Loci VNTR polymorphisms (Variable Number of Tandem Repeats, MLVA; Whatmore et al., 2016; Vergnaud et al., 2018). Robust phylogenetic relationships can be obtained from nucleotide sequencing data owing to the strictly clonal evolution of classical *Brucella* spp. (Whatmore and Foster, 2021). The first MLST scheme included nine loci, seven housekeeping genes, the outer membrane protein gene *omp25* and int-hyp. Six *B. abortus* MLST9 sequence types (STs) were initially described (Whatmore et al., 2007). Twenty-six *B. abortus* MLST9 STs are recorded in the current version of the *Brucella* spp. MLST database.^1^ Including 12 housekeeping genes in the genotyping resulted in the more discriminatory MLST21. Thirty MLST21 STs were initially described in 172 *B. abortus* strains defining three clades (A, B, C including C1 and C2; Whatmore et al., 2016) and 43 STs are recorded in the current version of the *Brucella* spp. MLST database (see text footnote 1). The rare clades A and B include strains originating almost exclusively from Africa and allow defining the most ancestral nodes within the *B. abortus* phylogeny. Clades C1 and C2 are found on five continents and their presence in Africa seem to result from livestock importation (Whatmore et al., 2016; Vergnaud et al., 2018). Several MLVA genotyping schemes have been proposed for *Brucella*, one most commonly used scheme is MLVA16 (Scholz and Vergnaud, 2013). MLVA16 combines two panels of markers: one VNTR panel with a low discriminatory ability allows determining the species and main genetic lineages, and the other VNTR panel with a high discriminatory ability but low phylogenetic value allows differentiating strains in local outbreaks (Scholz and Vergnaud, 2013; Whatmore and Foster, 2021). The current version (Brucella v4_6_5) of the Brucella MLVA data hosted by MLVAbank^2^ contains *in vitro* data derived from more than 1,400 *B. abortus* strains. The eight VNTR loci with the low discriminatory power (MLVA8) cluster *B. abortus* into 28 genotypes represented by at least two entries. MLVA alone is not suitable for phylogenetic reconstructions because of the high homoplasy at VNTR loci (Keim et al., 2004), but interestingly, MLVA using the low discriminatory markers (MLVA8, MLVA10 or MLVA11) and MLST clustering are highly congruent allowing to indirectly deduce phylogenetic signal from MLVA data (Vergnaud et al., 2018). Consequently, MLVA may constitute a first-line assay with low phylogenetic resolution until whole-genome sequencing (WGS) becomes sufficiently low-cost to be directly used as first line assay. MLVA genotypes can be readily deduced from WGS data with sequencing reads longer than 200 bp so that the highly discriminatory MLVA loci can also constitute a strain identity control tool (Vergnaud et al., 2018; Holzer et al., 2021; Pelerito et al., 2021).

The availability of whole-genome sequence (WGS) data opened the way to whole-genome and core genome MLST assays (wgMLST and cgMLST, respectively) with much higher resolution and phylogenetic value than these classical genotyping tools (Janowicz et al., 2018; Abdel-Glil et al., 2022). Genome-scale MLST assays as well as genome-wide SNP-genotyping confirmed the presence of the four major clusters A, B, C1, C2 in a collection of *B. abortus* with strains collected at a global scale (Whatmore and Foster, 2021; Abdel-Glil et al., 2022).

The current knowledge on the genetic diversity of *B. abortus* circulating in Kazakhstan is limited to MLVA-typing data. Interestingly, the MLVA genotyping investigations demonstrated low genetic diversity among strains circulating in Kazakhstan (Shevtsov et al., 2015; Daugaliyeva et al., 2018). Inclusion in the analysis of strains deposited since 1935 made it possible to describe the predominance of *B. abortus* clade C1, with genetic proximity of the majority of strains to Russian, Chinese and European strains. A few strains were assigned to *B. abortus* clade C2 (Shevtsova et al., 2016; Daugaliyeva et al., 2018). In order to better understand the population structure and origins of *B. abortus* in Kazakhstan, we selected 49 representative strains from Kazakhstan for whole-genome sequencing. We also selected 36 *B. abortus* strains representing the genetic diversity previously uncovered by MLVA within the historical collection maintained by Inrae, Nouzilly, France (Vergnaud et al., 2018) to determine phylogenic relations among *B. abortus* strains and to evaluate the usefulness of genomic data in the epidemiological control of the infection in a highly endemic region, such as Kazakhstan.

## 2. Materials and methods

### 2.1. *Brucella abortus* DNA and selection of strains for WGS

DNA analyzed were selected among 213 *B. abortus* strains isolated in Kazakhstan between 1943 and 2015 from bovine clinical material (aborted fetuses, lymph nodes, or whole blood). The strains were previously characterized by MLVA genotyping (Shevtsov et al., 2015; Shevtsova et al., 2016). The full MLVA assay (MLVA16) resolved 28 genotypes, 12 of which were represented by individual strains, the remaining genotypes were represented by up to 86 strains. The choice of strains for WGS was based on MLVA data, year and geographic origin. We selected 49 strains representing the 28 genotypes (up to eight strains per MLVA16 genotype). The selected strains originated from eight regions of Kazakhstan (Supplementary Table S1; Supplementary Figure S1).

Similarly, 212 *B. abortus* representative strains from the Inrae BCCN (Brucella Culture Collection Nouzilly) collected worldwide during the period 1976–2006 were previously characterized by MLVA (Vergnaud et al., 2018). Clade B and clade C represented 197 and 15 strains, respectively. A representative subset of 36 strains was selected for WGS (Supplementary Table S1).

### 2.2. Whole-genome sequencing

Kazakhstan strain sequencing was performed on the MiSeq platform (Illumina) as recommended by the manufacturer (Illumina, CA, United States). Nextera XT DNA Library preparation Kit (Illumina, CA, United States) was used to prepare libraries with double barcoding. Paired-end libraries were sequenced using MiSeq Reagent Kit v3 (600 cycles or 300 bp read length). *De novo* assemblies were produced using SKESA version 2.3.0 (Souvorov et al., 2018). The assemblies had an average of 49 contigs (range 33 to 88), an average N50 value of 171 kb (range 75 to 260 kb) and an average total assembly length of 3.25 Mb (range 3.229–3.256 Mb).

BCCN strains were sequenced by Genoscreen (Lille, France) using the MiSeq platform (Illumina). Read length was 250 bp. Sequencing reads were assembled using SPAdes version 3.13 (Bankevich et al., 2012). The assemblies had an average of 25 contigs (range 18 to 60), an average N50 value of 417 kb (range 251 to 884 kb) and an average total assembly length of 3.27 Mb (range 3.260–3.327 Mb).

Whole-genome MLST was run on genome assemblies using the BioNumerics “MLST for WGS” and “WGS tools” plugin and associated *Brucella* spp. scheme (Applied-Maths, Sint-Martens-Latem, Belgium). The plugins were also used to recover the MLST9 and MLST21 assignments (Whatmore et al., 2007, 2016) defined in the *Brucella* spp. database hosted by PubMLST (Jolley et al., 2018; Supplementary Table S1).

Raw reads were deposited in the European Nucleotide Archive BioProject PRJNA892249 (Kazakhstan collection) and PRJNA901374 (BCCN collection, France). Individual sequence reads archives (SRA), bioproject and biosample accessions are indicated in Supplementary Table S1.

### 2.3. Whole-genome single-nucleotide polymorphism analysis

All publicly available *Brucella* assemblies, sequence reads archive, and associated metadata, were downloaded from EBI ENA (read archives) or NCBI (assemblies) (last updated 2022-09-10). Sequence read archives were *de novo* assembled with SKESA. All assemblies were imported into BioNumerics version 8.1 (Applied-Maths, Sint-Martens-Latem, Belgium). The assemblies were used to produce artificial reads data sets (50 bp long, 10x coverage) for SNP calling by read mapping using genome assembly GCA_000740155 (*B. abortus* strain Tulya) as reference genome (Blouin et al., 2012; Vergnaud et al., 2018). The BioNumerics parameters for reads mapping were 95% minimum sequence identity, minimum inter-SNP distance 12 base-pairs. Maximum parsimony trees were drawn within BioNumerics. The list of all public datasets evaluated is presented in Supplementary Table S2. Supplementary Table S2 includes comments facilitating the selection of strains, including “duplicates” (more than one dataset available for the same strain), “redundant” (coincident wgSNP genotype). Some datasets provided poor coverage or induced topological issues, due to sample mix or inappropriate assembly procedure.

For input in BEAST version 1.10.4 (Suchard et al., 2018), a table of SNP positions from selected strains was exported from BioNumerics and SNPs were concatenated as fasta files. BEAST was run under a general time-reversible model of nucleotide substitution with a gamma distribution between sites, a relaxed molecular clock, Bayesian skyline plot (BSP) demographic model, lognormal distribution for population sizes as previously described (Kamath et al., 2016). The convergence of 20 independent runs with a chain length of 150 million was examined using Tracer v1.7 (Rambaut et al., 2018). The selected runs were merged with a burning of 15 million using LogCombiner and TreeAnnotator (Suchard et al., 2018). The resulting trees were visualized using FigTree v1.4.4 (Rambaut, 2018).

### 2.4. Analysis of homoplasia in MLVA profiles

The previously published MLVA16 profiles of 49 strains from Kazakhstan were used within BioNumerics for clustering based on categorical data distance measure and unweighted paired group with arithmetic means method (UPGMA). MLVA homoplasia was recognized when MLVA clustering was not congruent with the phylogeny deduced from wgSNP analysis.

## 3. Results

### 3.1. MLST assignment of the 49 *Brucella abortus* strains from Kazakhstan

Four MLST9 STs were present, ST1 (two strains) ST2 (45 strains), ST5 (one strain), and ST119 (new MLST9 genotype, represented by one strain). ST2 and the closely related ST119 belong to subclade C1 whereas ST1 and ST5 belong to subclade C2 (Whatmore et al., 2007). MLST21 resolves one additional genotype, as subclade C1 strain Kaz041 defined a new allele at MLST21 locus csdb due to a single-nucleotide variation (Supplementary Table S2). Using the wgMLST analysis assay, alleles could be called at 3,312 up to 3,345 loci in the 46 clade C1 strains. Seven hundred and three loci were called in all 46 strains and were polymorphic. Supplementary Figure S1 shows the clustering derived from the corresponding wgMLST character dataset of the 46 clade C1 strains.

### 3.2. Wgsnp based phylogenetic analysis of the 46 *Brucella abortus* subclade C1 strains from Kazakhstan

**Figure 1:**
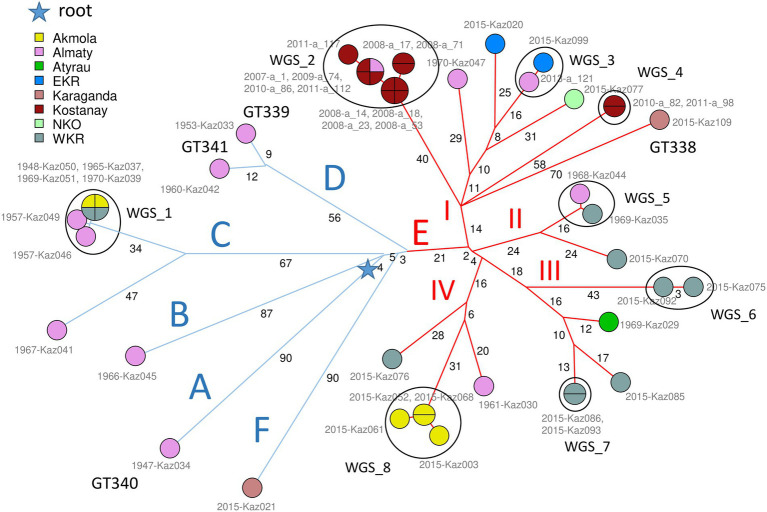
*Brucella abortus* subclade C1, 46 strains from Kazakhstan. Maximum parsimony tree based on core genome SNPs. 1,146 SNPs were called by mapping on genome accession GCA_000740155 (*B. abortus* clade B strain Tulya). The size of the resulting tree is 1,151 SNPs (homoplasia 0.44%). Thirty-three whole-genome SNP (wgSNP) genotypes are resolved. Branch lengths of two SNPs and more are indicated by black numbers. Strains are labeled in gray with collection year and strain Ids and colored according to region of origin as indicated. The MLVA11 genotype is indicated for new lineages distinct from GT72 (GT338 to GT341). The blue star indicates the root of the phylogeny (branching point toward *B. melitensis* type strain 16 M used as outgroup). From the blue star, early splits define six branches, labeled A to F. Blue branches A to D are defined by a few ancient strains isolated between 1947 and 1970. Blue branch F is defined by one recent strain, KAZ021 isolated in 2015. Red branch E with 34 strains (24 wgSNP genotypes) is remarkable by its diversity (24 wgSNP genotypes) and high number of associated strains (34 out of 46). It contains all but one of the recent strains (isolated in 2007–2015) together with five ancient strains. The E branch is structured into four subbranches labeled I to IV in red. Strains closely related or coincident in terms of wgSNP genotype define eight clusters labeled WGS_1 to WGS_8.

**Figure 2:**
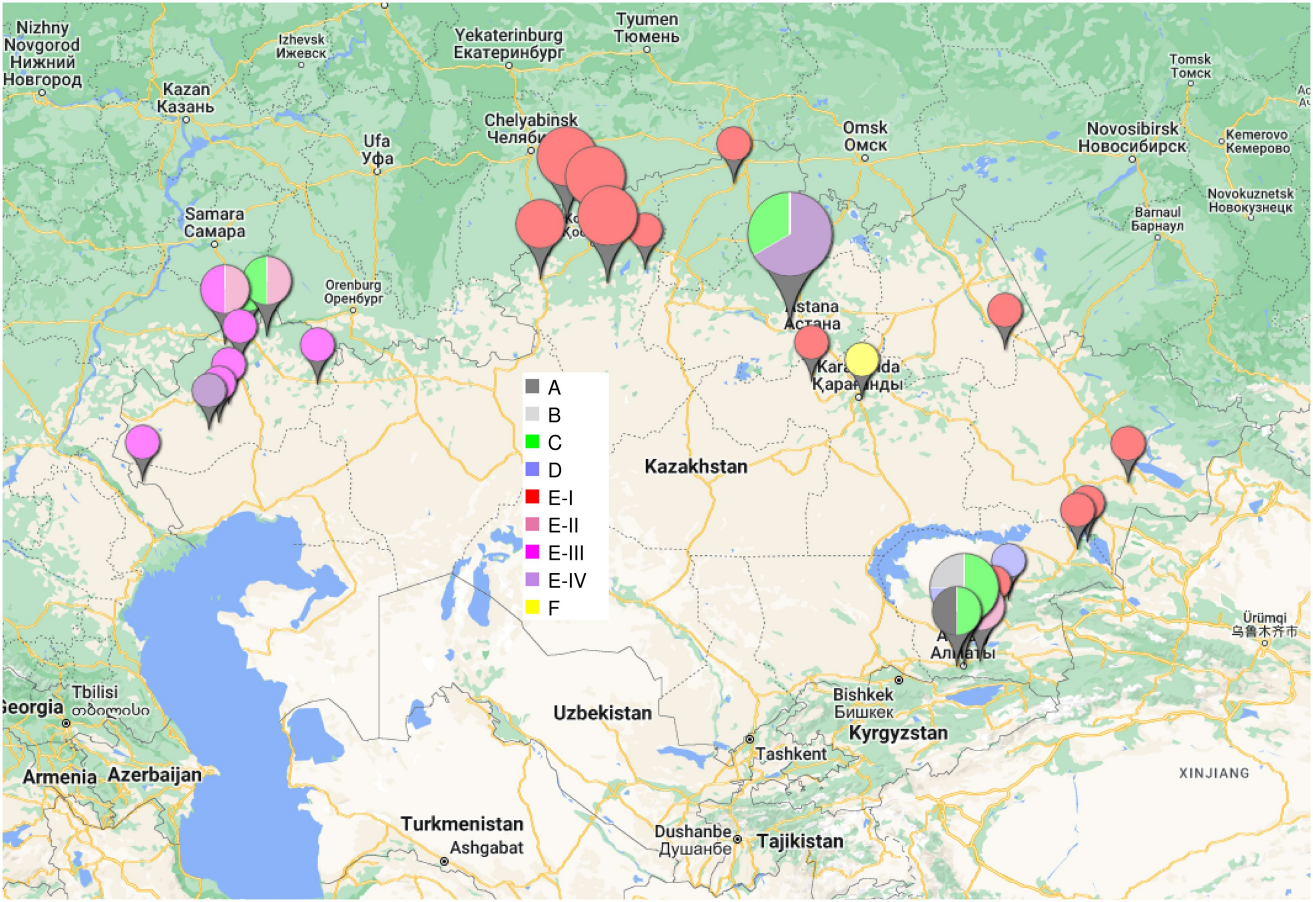
Geographic origin of the *B. abortus* clade C1 strains from Kazakhstan. The size of the labels reflect the number of strains (from 1 to 10) in the corresponding location. The color code shown in the central inset reflects the phylogenetic position (branch assignment) indicated in Figures 1, 3.

Figure 1 shows the phylogeny of 46 *B. abortus* subclade C1 strains from Kazakhstan deduced from wgSNP analysis. The maximum distance between two strains was 208 SNPs. Distances from root (blue star) to tips varied from 76 up to 117 SNPs. The analyzed Kazakhstan strains partitioned into six main branches labeled A to F. The topology obtained with wgSNP and wgMLST (Supplementary Figure S1) were identical. Branches A to D are defined by one to three different wgSNP genotypes corresponding to “historical” strains mainly collected in the Almaty region during the 1948–1970 period. All strains isolated from 2007 to 2015 were assigned to branches E and F. Whereas branch F was defined by a single strain, Kaz021, branch E could be further subdivided into four sub lineages labeled I to IV. Each one contained one or two historical strains in addition to recent strains. Lineage E-I is geographically associated with the Eastern and Northern parts of Kazakhstan (Figure 2). Lineages E-II to E-IV are predominantly associated with strains collected in 2015 in six settlements of the West Kazakhstan region (WKR).

### 3.3. Congruence between WGS phylogeny and MLVA clustering

The wgSNP analysis grouped 31 strains from Kazakhstan into 8 clusters labeled WGS_1 to WGS_8 in Figure 1; Supplementary Figure S1, which show a difference within each cluster of no more than 7 SNPs. Cluster WGS_1 comprised six “historical” strains lacking accompanying epizootological data, isolated in WKR, Akmola and Almaty regions between 1948 and 1970. The four strains identical by wgSNP from WGS_1 were also identical by MLVA16, while Kaz049 and Kaz046 differed at the highly variable Bruce07 locus. In spite of their very high wgSNP similarity, the strains were collected over a period of 13 years in three regions of Kazakhstan. Previous reports described similar long term maintenance of highly similar wgSNP or wgMLST genotypes (Garofolo et al., 2017; Janowicz et al., 2018; Allen et al., 2020; Holzer et al., 2021). Cluster WGS_2 was formed by ten strains isolated from Kostanay region in the 2008–2011 period and strain a_1 isolated in Almaty region in 2007. According to epidemiological data, nine out of ten strains from the Kostanay region represented four independent outbreaks. These strains represented three MLVA16 genotypes with a difference in the Bruce09 hypervariable locus. Cluster WGS_3 included two epidemiologically unrelated strains from EKR and Almaty regions, differing from each other by one SNPs, and by MLVA16 at the Bruce09 locus. Cluster WGS_4 was represented by two strains from the Kostanay region from two independent outbreaks which are identical in wgSNP and MLVA16. Cluster WGS_5 cluster was represented by two “historical” epidemiologically independent strains from WKR and Almaty regions identical in MLVA16 analysis and separated by two SNPs. Cluster WGS-6 combined two epidemiologically unrelated strains which are identical in their MLVA16 profiles and differ by three SNPs. Cluster WGS_7 combined two strains from the same outbreak in WKR, with an identical wgSNP genotype and differing in MLVA16 at locus Bruce07. Cluster WGS_8 combined four strains isolated in 2015 in the Akmola region, all from the same settlement and representing a single outbreak. The maximum difference between these strains is two SNPs, with all having an individual MLVA16 profile that differs in Bruce07 or Bruce09.

According to clustering on the basis of MLVA data, 10 MLVA16 genotypes (out of 28 genotypes) combined two to eight strains (Supplementary Figure S2). Homoplasia was suspected in five genotypes: MLVA_6 genotype includes two historical strains from Almaty (Kaz030 and Kaz041) and one recent strain from WKR (Kaz086). Kaz041 belongs to branch C, whereas Kaz086 and Kaz030 belong to E-III and E-IV, respectively, (Figure 1; Supplementary Figure S1). Genotype MLVA_7 included two strains, Kaz052 and Kaz070 isolated from Akmola and WKR regions in 2015, and belonging to E-IV and E-II, respectively. Genotype MLVA_14 combined four historical strains isolated from WKR and Akmola part of cluster WGS_1 from branch C and strain a_112 isolated in Kostanay in 2011 from branch E-I. Genotype MLVA_16 combined seven strains from cluster WGS_2 in branch E-I isolated in Almaty and Kostanay between 2007 and 2011, and also historical strain Kaz046 isolated in Almaty in 1957, belonging to cluster WGS_1 branch C. The MLVA_19 genotype combined strain Kaz020 (branch E-I, isolated in 2015, EKR region) and historical strain Kaz029 from Atyrau region from branch E-III. These lack of congruence between MLVA16 typing and wgSNP phylogeny are due to minor variations in the most variable loci constituting panel2B, in agreement with previous observations regarding the instability of these loci (Garcia-Yoldi et al., 2007; Maquart et al., 2009; Allen et al., 2020).

### 3.4. Comparison of the 46 *Brucella abortus* subclade C1 strains from Kazakhstan with public datasets

**Figure 3:**
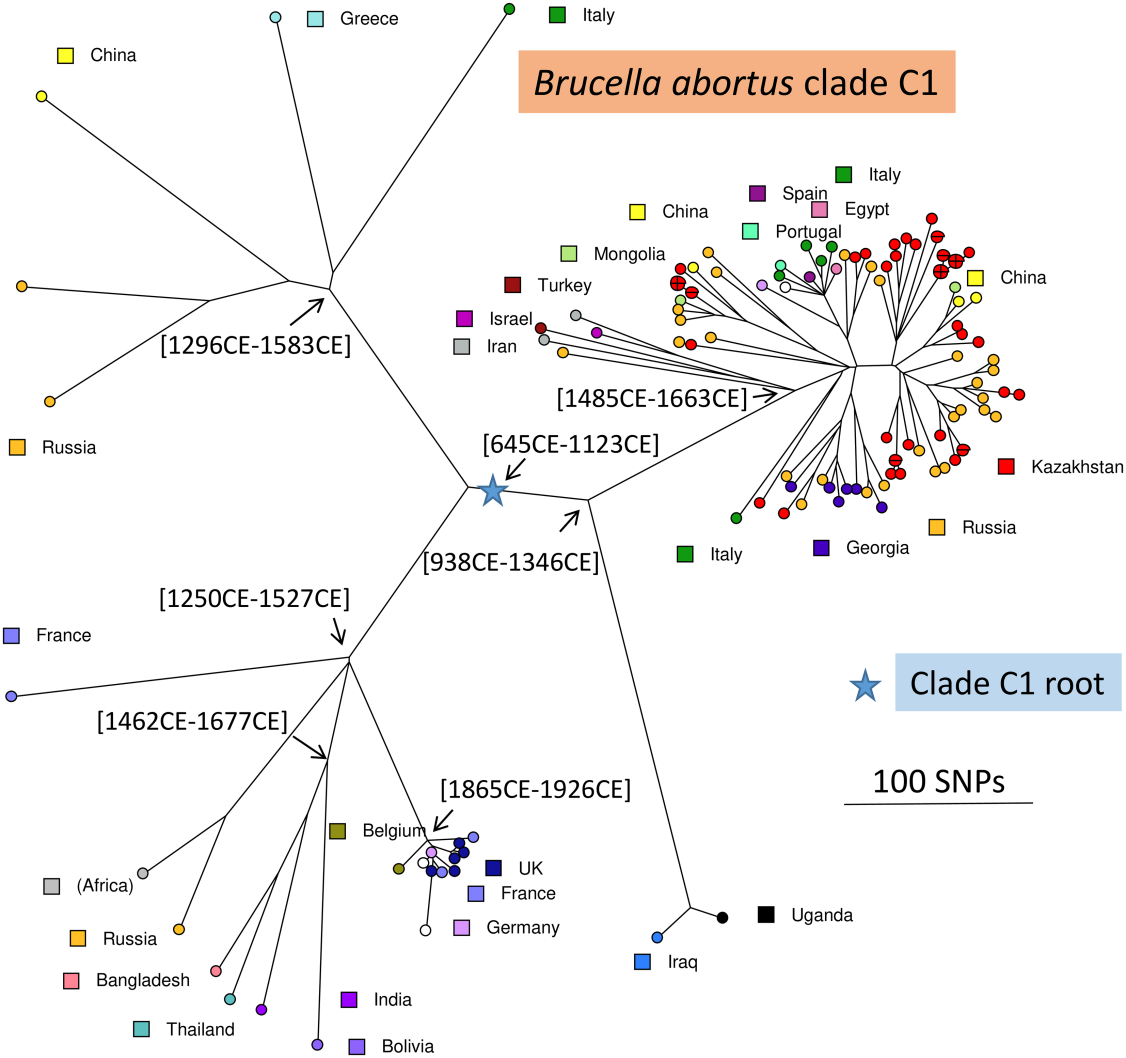
*Brucella abortus* subclade C1, position of the Kazakhstan strains within the global subclade C1 phylogeny. Maximum parsimony tree based on core genome SNPs. A total of 123 strains was used, including 77 selected strains of worldwide origins in addition to the 46 Kazakhstan strains. 5,446 SNPs were called by mapping on genome accession GCA_000740155 (*B. abortus* strain Tulya). The size of the resulting tree is 5,487 SNPs (homoplasia 0.75%). Strains are colored according to country of origin as indicated. The blue star indicates the root of the phylogeny (branching point toward *B. abortus* clade B strain Tulya used as outgroup). Representative estimated divergence dates are indicated (CE, common era).

**Figure 4:**
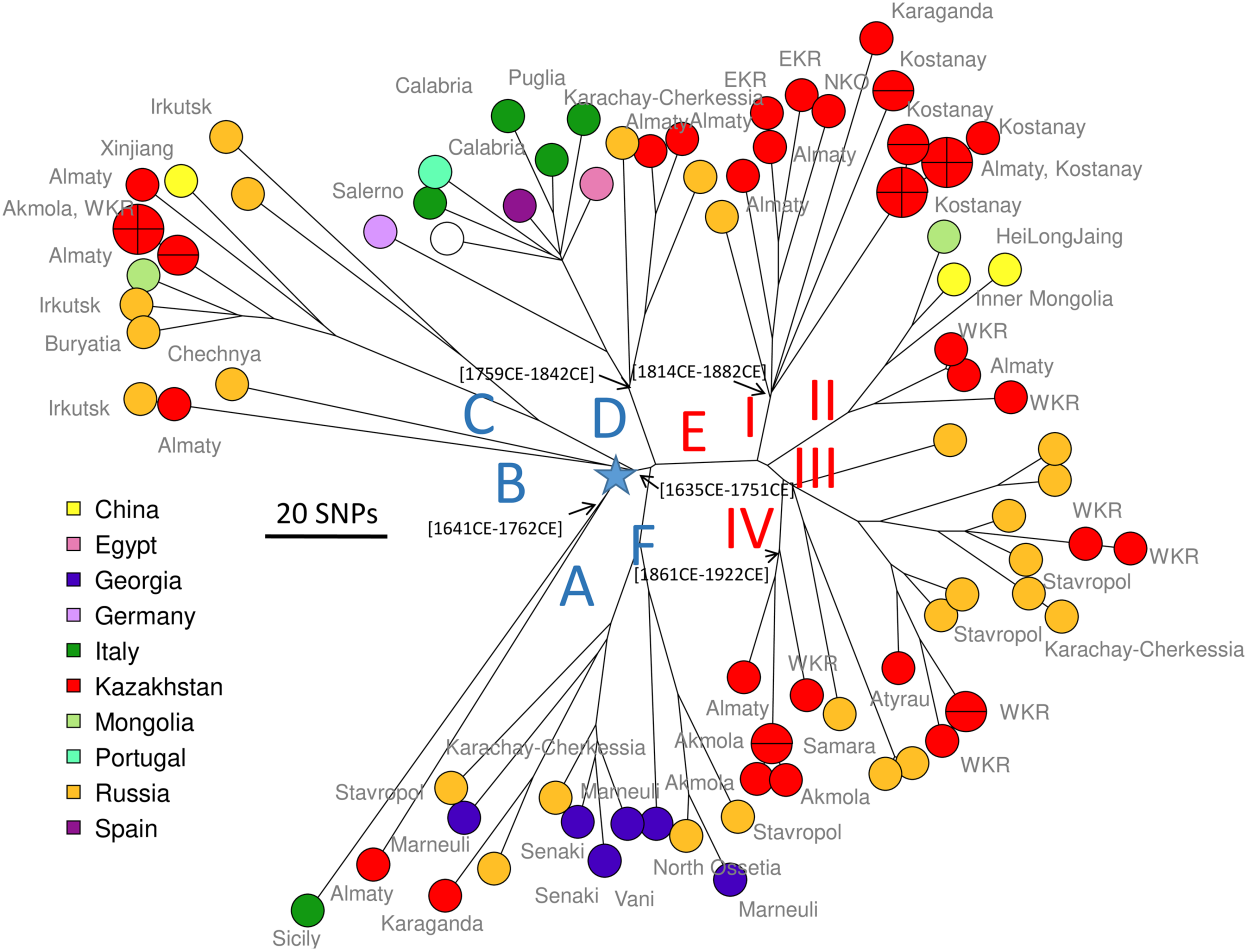
Zoom on *B. abortus* subclade C1, position of the Kazakhstan strains within the global subclade C1 phylogeny. Close-up on Figure 3. Strains are colored according to country of origin as indicated and labeled with region of origin when known. The blue star indicates the root of the phylogeny (branching point toward *B. abortus* clade B strain Tulya used as outgroup). Branch names A to F defined in Figure 1 are shown. Representative estimated divergence dates are indicated (CE, common era).

For comparison, we recovered 148 public WGS datasets, and eight *B. abortus* subclade C1 WGS datasets from the BCCN collection (Supplementary Table S2). Fifty-three duplicate datasets were removed (WGS data available as assembly and sequence reads archives, or identical strains sequenced by different institutions). Datasets contributing terminal branches shorter than five SNPs were also removed. Figure 3 shows the result of wgSNP analysis of the 46 *B. abortus* subclade C1 strains from Kazakhstan together with 77 selected WGS datasets of worldwide origins. Starting from the root, four main lineages were defined. Branch length from root to tip vary from 280 up to 309 SNPs. All 46 Kazakhstan strains clustered in one of these four clade C1 sub-lineages. Most Kazakhstan strains were closest to strains from neighboring countries, Russia and to a lower extend, China. About 10 independent introductions from these neighbors would be sufficient to explain the observed topology of the tree. Figure 4 shows an enlarged view of the subclade C1 lineages containing the Kazakhstan strains, together with the region of origin of the strains. Most strains from Kazakhstan appeared to be closest to strains from neighboring Russia and Georgia (Sidamonidze et al., 2017; Kovalev et al., 2021). For instance, the unique branch F representative from Kazakhstan, Kaz021 isolated in 2015, is surrounded in Figure 4 by multiple strains from Russia (North Caucasus) and Georgia. Branches A and D showed interesting features. In branch D, a split created a sublineage populated by seven strains collected in Western Europe, and one from Egypt. Branch A comprised only one strain from Kazakhstan and one from Italy.

### 3.5. Comparison of the three *Brucella abortus* clade C2 strains from Kazakhstan with public datasets

Public databases contain 880 *B. abortus* subclade C2 WGS datasets including assemblies and sequence read archives (Supplementary Table S2). After removal of datasets achieving a poor coverage of the reference genome, 870 datasets were available for comparison. Strain Kaz031 from Kazakhstan clustered in a tight group of approximately 100 strains, including representatives of strains 2308, RB51, S19, and *B. abortus* reference strain 544. RB51 is used as a vaccine, and was derived from 2308 (Schurig et al., 1991). Of note, Kaz031 differs by two SNPs from *Brucella* vaccine strain A19 (assembly accession GCA_003290345). The two other subclade C2 strains from Kazakhstan clustered together with vaccine strain 104 M. A total of 80 and 87 SNPs separated strains Kaz027 and Kaz025 from the vaccine strain *B. abortus* 104 M (assembly accession GCA_001296965). The two strains are also separated by three up to 18 SNPs from assembly accessions GCA_000250835 (an entry incorrectly labeled as a derivative of *B. melitensis* type strain 16 M, strain 16M13W), GCA_000292025 and GCA_000298635 (Supplementary Figure S3).

### 3.6. Global view of *Brucella abortus* phylogeny based on currently available WGS datasets

**Figure 5:**
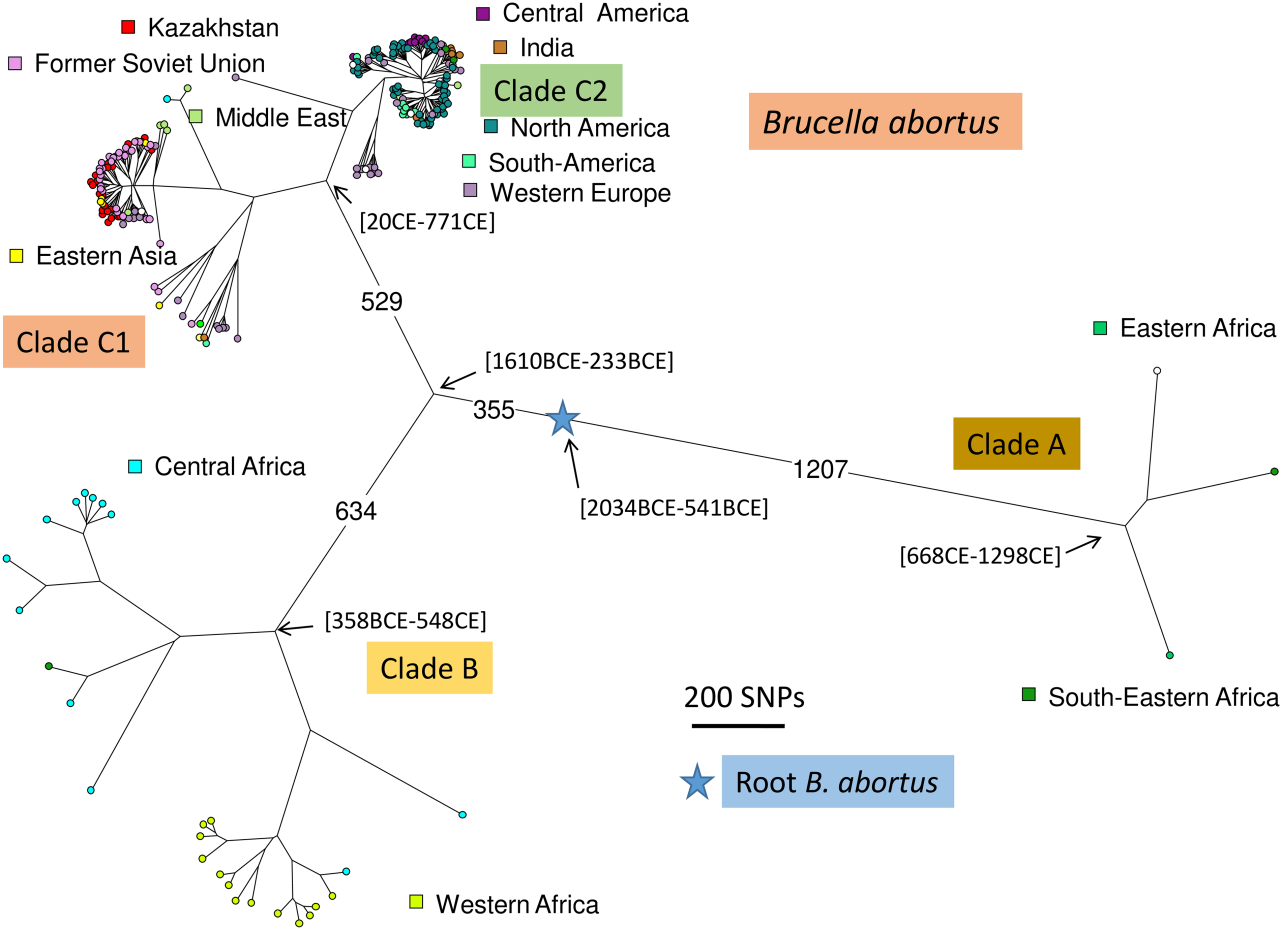
Global *B. abortus* phylogeny. Maximum parsimony tree based on core genome SNPs. A total of 236 representative strains was used and 16,987 SNPs were called by mapping on genome accession GCA_000740155 (*B. abortus* strain Tulya). The size of the resulting tree is 17,178 SNPs (homoplasia 1.12%). Nodes are colored according to geographic origin as indicated. The blue star indicates the root of the phylogeny (branching point toward the *B. melitensis* type strain 16 M). The largest branch lengths and the scale are shown. Representative estimated divergence dates are indicated (CE, common era; BCE, before common era).

A total of 1,169 *B. abortus* WGS datasets could be investigated including public datasets (Supplementary Table S2) and the 85 datasets produced for this report. Two hundred and thirty-six datasets were kept after removal of duplicates, redundant datasets, poor datasets, and closely related strains contributing terminal branches shorter than 5 SNPs. Figure 5 shows a global view of the phylogeny of *B. abortus* deduced from this dataset. The three clades, A, B, and C, were clearly resolved. Clades A and B showed a strong geographic association, clade A with East Africa, and clade B with West Africa. Clade C subclade C1 was predominantly associated with the eastern part of Eurasia, and subclade C2 with the western part of Eurasia as well as North and South America. Estimated divergence dates of the most ancestral nodes are shown. Supplementary Figure S4 shows the same phylogeny with more detailed metadata information.

### 3.7. Tentative dating of *Brucella abortus* introduction events in Kazakhstan

As a preliminary attempt to date these introduction events, we applied BEAST to the previous selection of 236 *B. abortus* and to 193 *B. melitensis* strains (Supplementary Table S2). *B. melitensis* and *B. abortus* are closest relatives within genus *Brucella*. They share the same mutation inactivating the Entner–Doudoroff pathway (EDP) for hexose catabolism, suggesting that their most recent common ancestor was already an obligate animal pathogen (Machelart et al., 2020). The inclusion of *B. melitensis* allowed to take advantage of the WGS sequence data recovered from a well-dated Medieval sample (Sardinia, *circa* year 1,375 CE), thus providing a key time point (Kay et al., 2014). SNPs were called by mapping on genome accession GCA_000740155 from *B. abortus* strain Tulya (23,086 SNPs were identified). Representative dating estimates are included in Figures 3–5. The analysis suggested that currently circulating *B. abortus* strains in Kazakhstan originated from imports in the 19^th^ and 20^th^ century. The split toward the European sublineage in branch D would have occurred in year 1759–1842. The Italian and Kazakhstan strains constituting branch A would share a most recent common ancestor *circa* year 1,641–1762. The estimated molecular evolution rate is 0.3 SNP per year per genome (95% HPD—highest probability density—range 0.24–0.45). A higher evolution rate of 0.46 SNP per year per genome (95% HPD range 0.30–0.74) was previously proposed (Kamath et al., 2016). The main differences in the two investigations were the collection of strains used (both *B. abortus* and *B. melitensis* in the present investigation versus North American *B. abortus* clade C2 only in Kamath et al.) and the inclusion in the present investigation of the Medieval sample.

## 4. Discussion

Brucellosis in cattle remains a major problem for cattle breeders in Kazakhstan. Genetic monitoring of genotypic dynamics is not included in the infection control strategy at the state level, but is implemented in the country through scientific grants and is mainly based on MLVA technology. This is the first study to describe the genetic diversity of *B. abortus* isolated between 1947 and 2015 in the territory of Kazakhstan using genome-wide sequence data that confirmed the presence of the two major subclades C1 and C2. Clades A and B were absent, in agreement with the previously published MLVA clustering analysis (Shevtsova et al., 2016; Daugaliyeva et al., 2018) and well established very strong geographic association of clades A and B with East and West Africa, respectively, (Whatmore et al., 2016; Vergnaud et al., 2018).

In this study, in five among the 10 MLVA16 genotypes represented by at least two strains, unrelated wgSNP genotypes were observed. The high percentage of homoplasia in the VNTR analysis is primarily due to the targeted selection of strains with identical MLVA16 from outbreaks which are unrelated by epidemiological data, and to the high level of homoplasia associated with the most variable VNTR loci. Four out of five cases of MLVA16 homoplasia involved combinations of the “historical” and currently circulating strains. Therefore, MLVA16 homoplasia seems higher in strains collected over an extended period of time. Eight wgSNP clusters were identical in MLVA16 or differed only in one or two hypervariable loci (Bruce07 or Bruce09), generally supporting the MLVA clustering. Previous studies of *B. abortus* strains using whole-genome SNP analysis and MLVA demonstrated similar results in outbreak differentiation and detection of imported strains, with a conclusion of a need to use genome-wide SNPs for reliable phylogenetic inference (Garofolo et al., 2017; Allen et al., 2020; Suárez-Esquivel et al., 2020; Holzer et al., 2021). Comparable strain clustering for MLVA11 and wgSNP allows MLVA in combination with epidemiological data to be considered as a first choice of methods to select strains for WGS in highly endemic regions, but only WGS analyzes will provide sufficiently precise phylogenetic information and might progressively become the first choice methods if global sequencing costs keep decreasing.

The identification of very closely related wgSNP genotypes in different regions with a difference of isolation by several years indicates a long-term circulation of *B. abortus* genotypes in Kazakhstan. Also, identification of the same wgSNP genotype cluster in unrelated outbreaks in the same region, such as Kostanay, indicates circulation of infected animals between farms. Thus, uncontrolled cattle trade and movement, as well as keeping animals from different farms on the same pastures, is postulated to be among main factors in the spread of the cattle brucellosis infection in Kazakhstan (Syzdykov et al., 2014; Charypkhan et al., 2019).

The Bayesian phylodynamic approach suggested that *B. abortus* lineages currently circulating in Kazakhstan were introduced in the 19th-20th centuries from Europe, mainly from Russia (North Caucasia). It may be interesting to note that evolution of subclades C1 and C2 showed similarities in this respect. The import of clade C2 into the United States was dated to the end of the 17^th^ century (Kamath et al., 2016), with closest neighbor lineages corresponding to strains isolated in Western Europe.

The introduction of *B. abortus* clade C1 in Kazakhstan might have happened during periods of human migration and because of the importation of numerous livestock for breeding with native breeds of cattle. Migration processes to Kazakhstan from the territory of the Russian Empire, and later the USSR, began with the accession of the northern territories of Kazakhstan in the Russian Empire in the 1730s and continued until the 1970s (Qazaqstan_tarihy, 2018; Presidential_Library_RF, 2022). The imperial period was characterized by mass migration of peasants, whose migration had an impact on the livestock breeding system of the nomadic people. Migrations had a wave-like character and were associated with the abolition of serfdom and the resettlement of free peasants from the European part of Russia since 1861. In 1889 the law on resettlement provided land plots and loans to peasant settlers. Crop failure and famine in European Russia and the Stolypin reforms of 1906–1911 further stimulated these migrations (Rather and Abdullah, 2018). In 1897 Russians made up 12 percent of the total population of Kazakhstan, i.e., 600,000 inhabitants. From the end of the 19th century to 1916, about 1,400,000 European Russians arrived in the Kazakh steppes, making up 40% of the population of the steppe regions of Kazakhstan (Bell-Fialloff, 2016). There are no exact data on imported livestock for that period, however, there are some data indicating that people moved with their livestock, and original Kalmyk-cattle-breeds appeared in Kazakhstan along with immigrants from Voronezh, Stavropol, Astrakhan, and other provinces of southern Russia (Belmont, 1960). During this period, many Kazakhs were forced to rebuild their traditional way of life with the transition to agriculture and a semi-nomadic lifestyle due to the seizure of land in favor of settlers for farming and taking rangelands to graze Russian riches’ cattle (Sailaukyzy et al., 2018). In the structure of Kazakhs herds, the number of cattle had increased because of greater demand for cattle meat. At the end of the 19th and the beginning of the 20th century, selective transformation of aboriginal cattle began, for which a massive import of cattle from various regions of Russia was carried out. For example, to create the Alatau breed from 1904 to 1940, Swiss and Kostroma cattle were brought to Kazakhstan and Kyrgyzstan from the Smolensk, Sumy and Kostroma regions (Soldatov, 2001). The Aulieatinskaya cattle breed has been developed since 1885 by crossing the Dutch Black Pied breed with aboriginal cattle and subsequent improvement in the 1930s of the 20th century by East Friesian bulls (Dmitriev and Ernst, 1989). All these processes might have constituted opportunities for a wide distribution of European strains of *B. abortus* in Kazakhstan, but it is important to note that essentially subclade C1 strains contaminate Kazakhstan whereas Western Europe is associated with subclade C2. Subclade C1 is common in North Caucasus (Kovalev et al., 2021). At the same time, the disastrous large-scale collectivization carried out in 1929–1933 resulted in a four-fold reduction in the local cattle population (Zhumasultanova, 2021), which could lead to a reduction in the historically circulating strains of *B. abortus*. The observed reduction in the genetic diversity of *B. abortus* strains in Kazakhstan in the 21st century is possibly associated with the successful implementation of epidemic-control activities similar to methods implemented in the 1970–1980s. During 4 years, from 1981 to 1985, the incidence of brucellosis in cattle decreased from 3.5 to 2.2% and a reduction in the most affected areas was as high as 30% (Shablov et al., 1988). Another factor for consideration is a two-fold reduction in the cattle population from 1991 to 2000.

The isolation of two *B. abortus* subclade C2 strains Kaz025 and Kaz027 genetically closest to the *B. abortus* 104 M vaccine strain is intriguing. We could not find records of the use of this vaccine in Kazakhstan. The *B. abortus* 104 M strain was first isolated from an aborted fetus of cattle in the central European Russia in 1929, selected and proposed as a vaccine strain for human vaccination by Kh. S. Kotlyarova in 1950 (Kotlyarova, 1950; Shumilov et al., 1983). Despite proven immunogenicity, strain 104 M did not find wide application in the USSR and was used in experimental vaccination of cattle and in limited production trials on small cattle. In this connection, the probability of importation of a vaccinated animal is low. We are more inclined toward the introduction of genetically close pathogenic strains. Expanding the results of genome-wide data on *B. abortus* strains isolated in the central European Russia will improve understanding of the origin of strains Kaz025 and Kaz027.

## 5. Concluding remarks

The new data of various origins contributed in this report strengthen the strong association of clades A and B with East and West Africa, respectively. The topology of the observed phylogeny combined with human history is pointing to East Africa as current most parsimonious scenario for the origin of *B. abortus*. The WGS data analysis of *B. abortus* strains from Kazakhstan shows that currently circulating lineages were introduced only recently in Kazakhstan, most of them during the 19^th^ or 20^th^ century. The closest currently known lineages are present in Caucasia, in agreement with the history of recent population migrations. This recent introduction is reminiscent of the situation described in Costa Rica, in which WGS data analysis allowed to identify five independent introductions responsible for the current population structure of *B. abortus* in Costa Rica (Suárez-Esquivel et al., 2020). Costa Rica was contaminated by *B. abortus* subclade C2 strains, imported from neighboring countries, whereas Kazakhstan was contaminated essentially by subclade C1 strains. This reflects the progressive spread of *Brucella* worldwide.

## Data availability statement

The datasets presented in this study can be found in online repositories. The names of the repository/repositories and accession number(s) can be found below: Bioproject PRJNA892249 and PRJNA901374.

## Author contributions

GV editing of the original manuscript. GV, AC, and AShe conceptualization, designed the experiments, and analyzed the data. AShe and AC wrote the first draft of the manuscript. KB, MZ, ES, AShu, AA, DK, and YR genotyping and wrote sections of the manuscript. TK bacteriological researches and wrote sections of the manuscript. All authors contributed to the article and approved the submitted version.

## Funding

This study was supported by the Ministry of Education and Science of the Republic of Kazakhstan (grant no. AP08052352) and by the french «Agence Nationale de la Recherche» grant ASTRID430 Maturation ANR-14-ASMA-0002-02.

## Conflict of interest

The authors declare that the research was conducted in the absence of any commercial or financial relationships that could be construed as a potential conflict of interest.

## Publisher’s note

All claims expressed in this article are solely those of the authors and do not necessarily represent those of their affiliated organizations, or those of the publisher, the editors and the reviewers. Any product that may be evaluated in this article, or claim that may be made by its manufacturer, is not guaranteed or endorsed by the publisher.

## Footnotes

^1^https://pubmlst.org/organisms/brucella-spp/ (Accessed January 24, 2023).

^2^ https://microbesgenotyping.i2bc.paris-saclay.fr/
