# Supplementary figures and images for: Brucella abortus in Kazakhstan, population structure and comparison with worldwide genetic diversity
Source: Front Microbiol. 2023 Mar 22;14:1106994. doi: 10.3389/fmicb.2023.1106994 (PMC10073595; doi:10.3389/fmicb.2023.1106994)

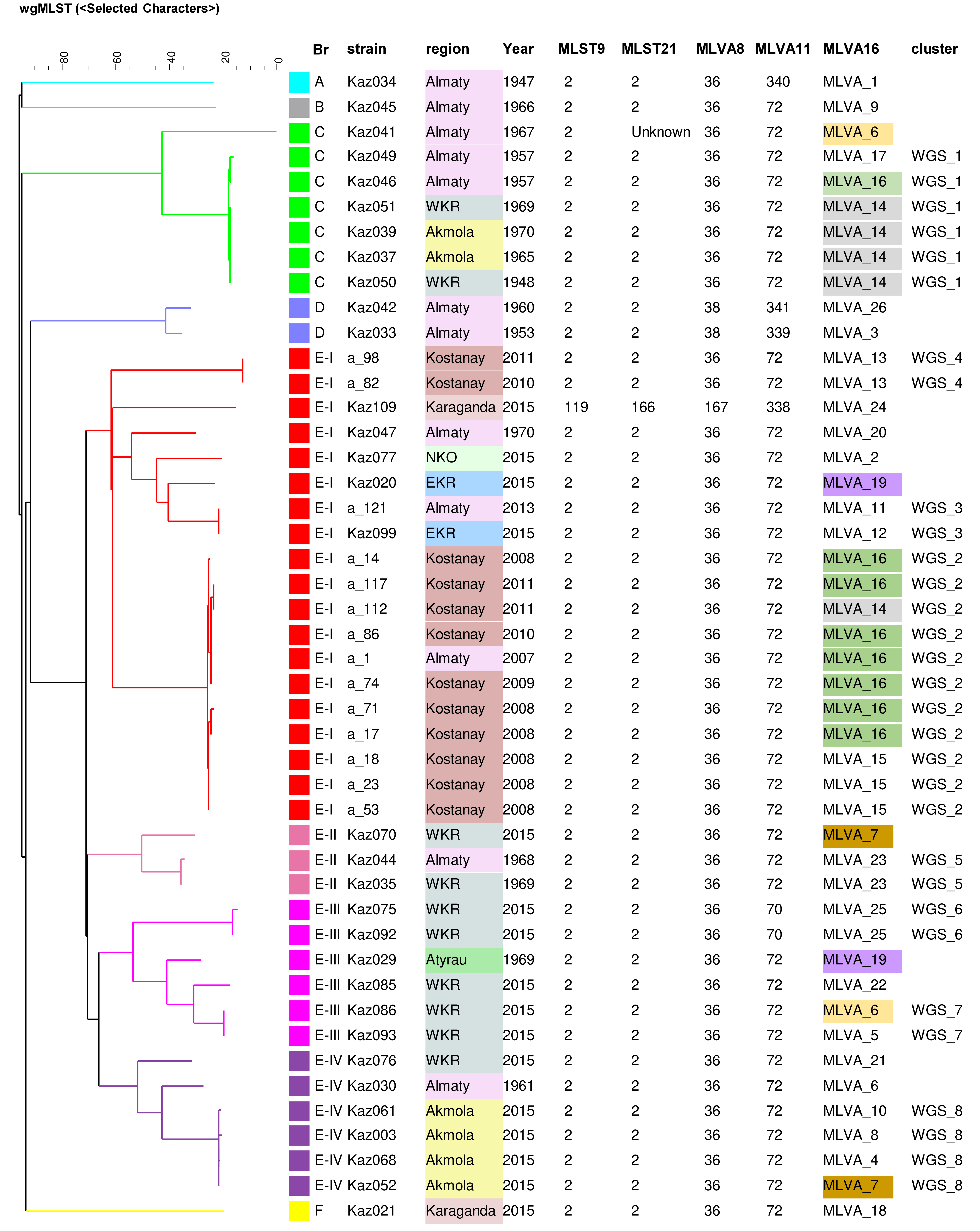

Supplement: Supplementary file 1 [file Image_1.TIFF]

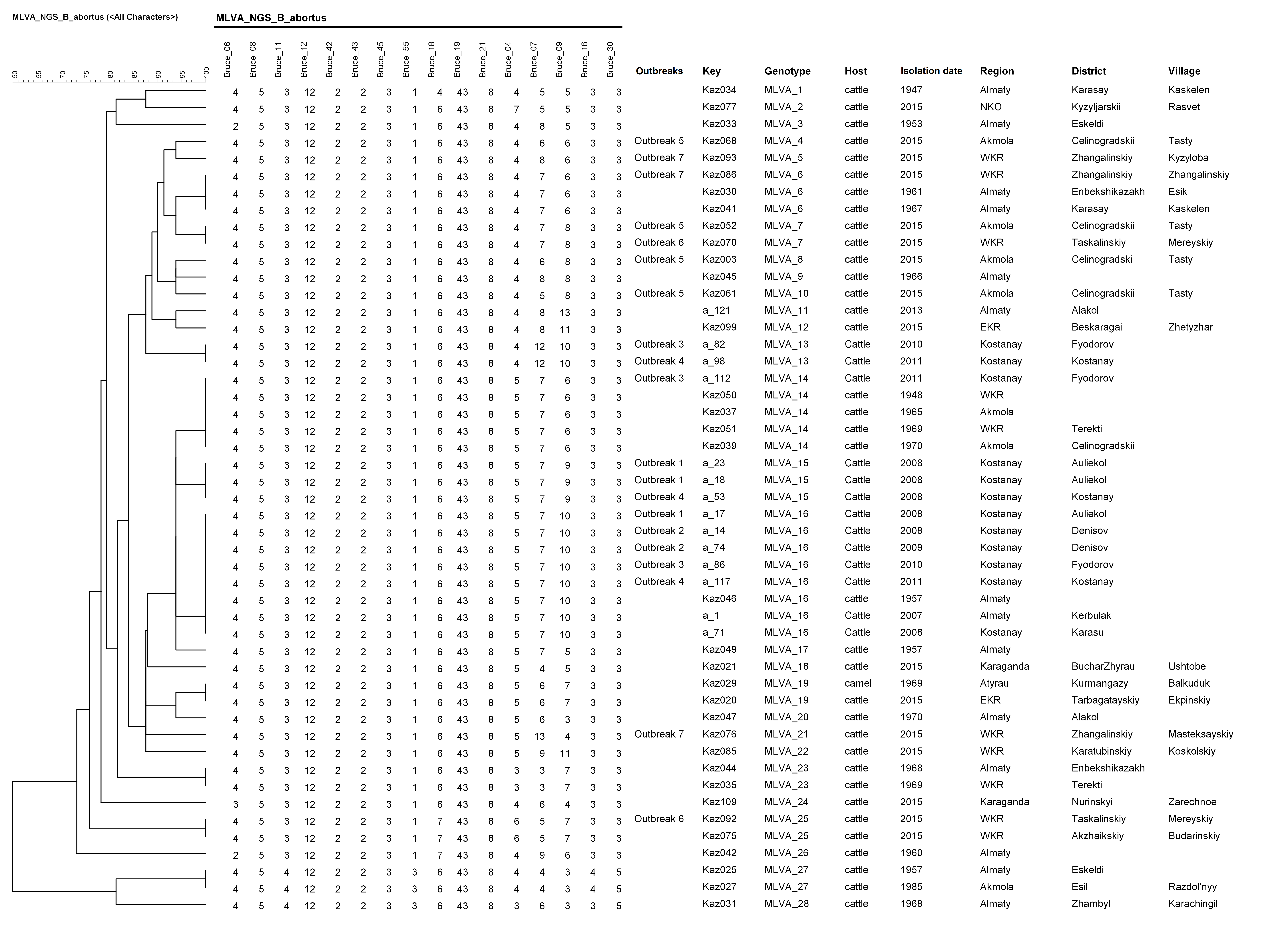

Supplement: Supplementary file 2 [file Image_2.TIFF]

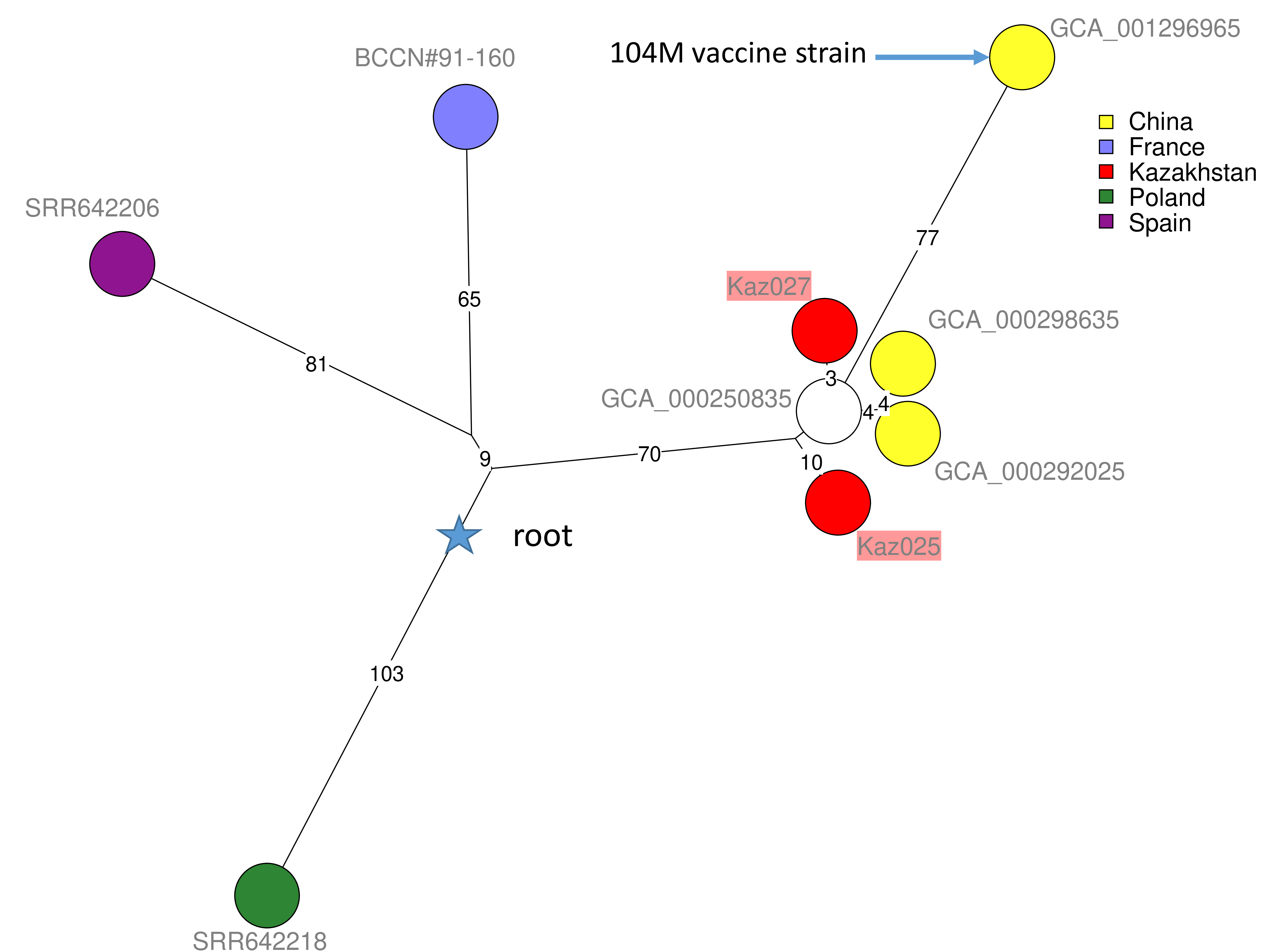

Supplement: Supplementary file 3 [file Image_3.TIFF]

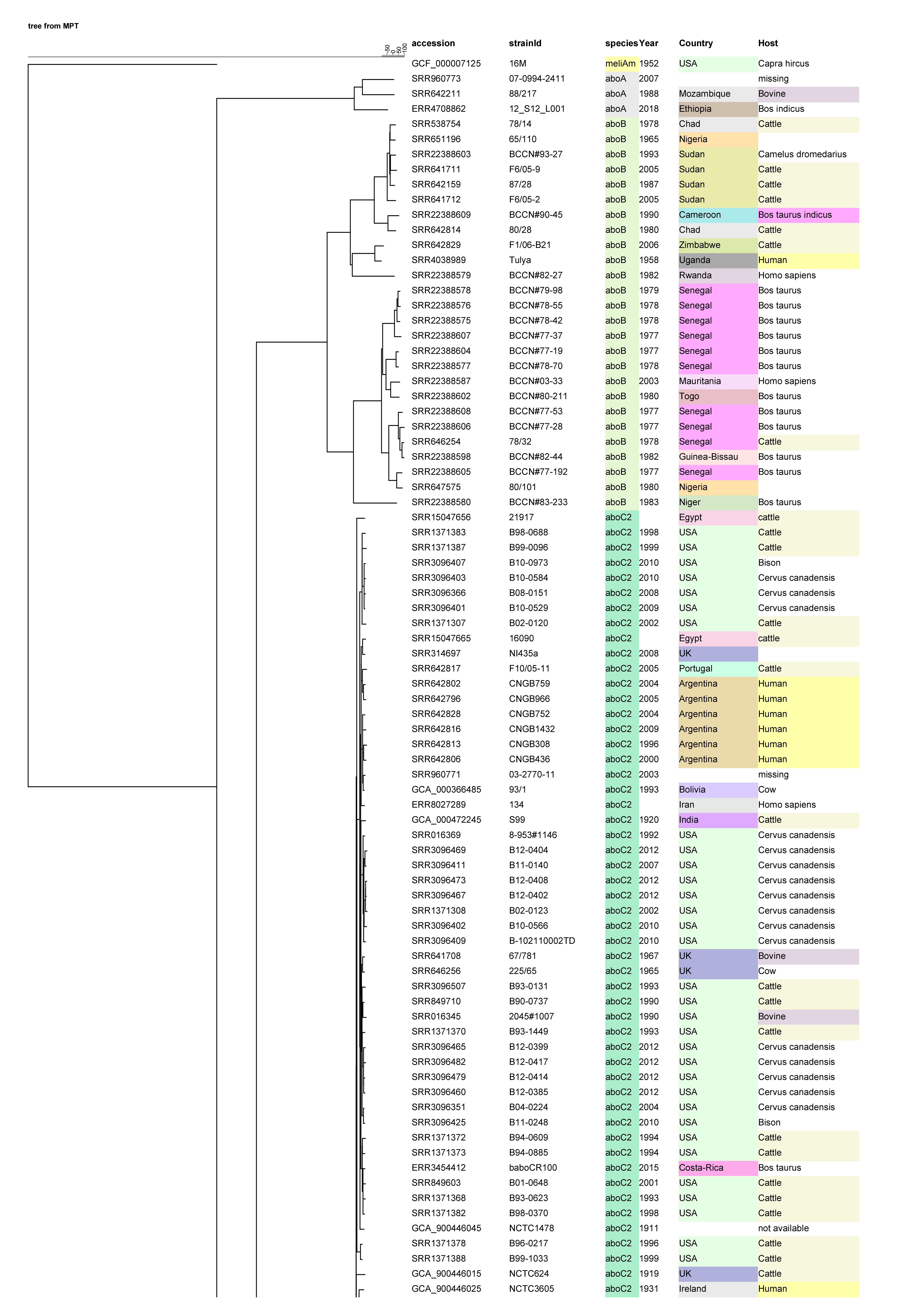

Supplement: Supplementary file 4 [file Image_4.TIFF]
